# Growth resilience to weather variation in commercial free-ranging chickens in Ethiopia

**DOI:** 10.1186/s12864-025-11561-6

**Published:** 2025-04-14

**Authors:** Georgios Banos, Mekonnen Girma, Bersabhe Solomon, Pourya Davoudi, Wondmeneh Esatu, Tadelle Dessie, Androniki Psifidi, Kellie Watson, Olivier Hanotte, Enrique Sánchez-Molano

**Affiliations:** 1 Centre for Tropical Livestock Genetics and Health (CTLGH), Scotland’s Rural College, Animal and Veterinary Sciences, Easter Bush, Midlothian, EH25 9RG UK; 2 Centre for Tropical Livestock Genetics and Health (CTLGH), ILRI, P.O. Box 5689 Addis Ababa, Ethiopia; 3 Centre for Tropical Livestock Genetics and Health (CTLGH), Roslin Institute, University of Edinburgh, Easter Bush, Midlothian, EH25 9RG UK; 4 Royal Veterinary College, University of London, London, NW1 0TU UK; 5 The Roslin Institute and R(D)SVS, University of Edinburgh, Easter Bush Campus, Midlothian, EH25 9RG UK; 6 School of Life Sciences, University Park, University of Nottingham, Nottingham, NG7 2RD UK

**Keywords:** Chicken, Sub-Saharan Africa, Weather resilience, Climate change, Growth, Genomics

## Abstract

**Background:**

The poultry industry in sub-Saharan Africa is a rapidly developing sector mostly based on smallholder farming. Increased demand for poultry-derived products, driven by the growing economy and population, has intensified importations of highly productive exotic breeds and crossbreeding with local ecotypes. However, commercial chickens with exotic genes often struggle to adapt to the local climate under smallholder farmers management. Understanding the chicken response to weather changes is crucial for developing selection schemes that ensure proper adaptation. In the present study, we derived individual phenotypes for growth resilience of commercial free-ranging chickens to changing weather conditions in Ethiopia. In addition, we performed genomic association analyses to assess the genetic background of these phenotypes and identify potential candidate genes of interest.

**Results:**

Novel resilience phenotypes describing changes in chicken growth profiles in response to weather fluctuation were developed. Variations in daily air temperature, relative humidity and amount of precipitation had the strongest impact on growth. Significant genomic variance was detected for growth resilience to changes in air temperature measurements and a temperature-humidity index. Genomic markers correlated with these resilience traits were mostly located within or near candidate genes associated with lipid metabolism and adipocyte homeostasis. Some of these genes have been previously linked to animal responses to environmental stressors in other species.

**Conclusions:**

The phenotypes of growth resilience of chickens to changing weather conditions exhibited significant genomic variation. The outcomes of this study may facilitate the genomic selection of commercial chickens that are not only highly productive, but also capable of maintaining their production levels under varying weather conditions.

**Supplementary Information:**

The online version contains supplementary material available at 10.1186/s12864-025-11561-6.

## Background

The agricultural sector in sub-Saharan Africa employs about 64% of the labour force and contributes to a substantial proportion of the Gross Domestic Product [1]. Within this sector, the poultry sub-sector is characterised by a diverse array of production systems, ranging from small family farms to large-scale commercial enterprises. The sector plays a vital role, not only as a food protein source, but also as a driver of social development and income. Approximately 80% of the total poultry production is associated with smallholder farming [2] and around 70% of the total chicken population are of indigenous breeds and ecotypes, although these proportions are highly variable across different regions [3].

In Ethiopia, the current national poultry production sector comprises around 56 million birds producing 77,000 tons of meat annually, of which 88% emanate from smallholder farms where chickens range freely [4]. Although the majority of these chickens are of indigenous ecotypes, the use of commercial crosses and imported exotic breeds is rapidly increasing due to their higher productivity [5], currently accounting for about 18% of the reared population [6]. This increase is fuelled by an expanding economy, a rapidly growing consumer population, and urbanisation. Indeed, demand for chicken meat in Ethiopia is estimated to increase by 268% in the next 25 years [7], prompting considerable pressure on the existing production systems. However, imported exotic breeds may be maladapted to the new local conditions and environmental challenges [8]. Climate change is a key challenge whose effect in Ethiopia is expected to be critically evident in the near future, with average air temperatures potentially increasing by 2.9 degrees Celsius by 2050 [9], accompanied by intense and unpredictable fluctuation in weather events [10]. These changes constitute a serious environmental stressor on chicken growth and production capacity, and commercial crosses based on exotic genes may be particularly vulnerable.

Consequently, breeding goals and methods must evolve to ensure selected individuals are capable of coping with the new challenges and avoid losses in chicken numbers and productivity [11]. A recent study on Ethiopian indigenous chickens [12] highlighted their genetic resilience to diverse environmental factors including air temperature, rainfall and land cover, and linked candidate genes to genetic adaptation to local climatic challenges. Thus, there is scope to develop novel chicken resilience phenotypes to inform existing and future breeding [13] and conservation [14] programmes aiming to mitigate the effect of climate change.

The aim of the present study was to investigate the impact of changing weather conditions on chicken growth in a popular commercial cross reared in Ethiopia by (i) deriving and examining novel chicken growth resilience phenotypes to weather volatility and (ii) assessing the genomic architecture of these new phenotypic traits of chickens. We deployed random regression methods on longitudinal data to derive resilience phenotypes and used genotype-by-sequencing data and genomic association analyses to estimate genomic parameters and identify potential molecular markers and candidate genes associated with chicken resilience to weather.

## Material and methods

### Animal data

Individual data on 1,590 chickens were available for the present study. All chickens were T451A Sasso, which is a slow-growing commercial dual-purpose type suitable for smallholder village conditions [15], resulting from a cross between T44 males and S51A females [16–18].

Data collection had taken place under a previous study [16] at the International Livestock Research Institute (ILRI) poultry research facility in Addis Ababa, Ethiopia, between 2019 and 2021 (Table 1). In that previous study, chickens grew in four batches outdoors in specially designed paddocks emulating the free-ranging semi-scavenging conditions that prevail in smallholder farms [13]. This phase started at the chicken age of 56 days, which represents the average age when chickens are sold to village smallholders for rearing in free range and lasted eight weeks. Live body weight was measured in grams and recorded weekly on each individual chicken together with the corresponding tag identifier, date of measurement, batch number and sex of the bird.

**Table 1:** Summary of data used in the study

Batch number	Number of chickens	Recording start date	Recording end date	Local season
1	436	18/12/2019	12/02/2020	Bega-Belg
2	429	13/07/2020	07/09/2020	Kiremt
3	406	14/10/2020	09/12/2020	Bega
4	315	24/12/2020	18/02/2021	Bega-Belg

Local seasons pertain to the calendar months of weekly chicken body weight measurement and recording: Bega (October to January), Belg (February to May) and Kiremt (June to September)

For the purposes of the present study, average daily growth rate was calculated for each chicken and week based on the live body weight difference between two consecutive weeks divided by seven. Thus, seven weekly measures of daily growth rate were available for each chicken.

Chicken genotypes from low-pass whole-genome sequencing and imputation had been produced and quality assured in the previous study [16]. Briefly, individual blood samples of 100μl were collected from each chicken, preserved in absolute ethanol, transferred onto QIAcard FTA Elute Micro cards, and transported to Neogen Genomics (USA) for low-pass whole-genome sequencing (0.5X coverage). SNP imputation was conducted by Gencove (USA) using a reference panel of 583 high-coverage (30X) chicken genomes, including the studied Sasso type, mapped to the *Gallus gallus* 6 assembly (GRCg6a), with a panel density of approximately 29 million variants. After assessing the accuracy of imputation, which was greater than 90% [16], quality control removed samples with call rates less than 90%, Single Nucleotide Polymorphism (SNP) markers with minor allele frequencies less than 0.02, and SNPs located on the sex chromosomes. Linkage Disequilibrium pruning (r^2^ > 0.8) was implemented. Duplicate samples with an identity-by-state estimate greater than 98% were also removed. After quality control, the final dataset for the present study comprised 1,586 genotyped chickens and 2,940,003 SNPs across 33 autosomal chromosomes.

### Weather data

Daily records of maximum, minimum and average air temperature, relative humidity, and amount of precipitation for the period of study (2019–2021) were extracted from three main weather stations in Addis Ababa (Supplementary File 1) as well as satellites (NASA and Copernicus EU program) and radar [19].

Average daily air temperature and relative humidity records were combined to develop a temperature-humidity index (THI) using the Finocchiaro method [20]. THI was added to the list of weather variables.

The profile of each weather variable was explored by calendar year and the following three local seasons: Bega (from October to January), Belg (from February to May) and Kiremt (from June to September). Bega is often characterised by mild average and maximum daily air temperatures, relatively low minimum temperatures at night, and low humidity. The Belg season is characterised by variable humidity, cold nights, and mild daily average temperatures, but certain days tend to reach relatively high maximum temperatures. Kiremt is the wet season, with frequent heavy rain, high humidity, and relatively mild air temperatures.

Weekly means and variances of the daily values of each weather variable were calculated and matched to the corresponding weekly measurements of chicken daily growth for the ensuing data analyses.

### Population reaction norms at the phenotypic level

A regression model (1) was used to describe average daily growth change in response to changing weather, manifested separately in each weather variable, at the population level [21]:

1 $${y}_{ij}=X+f\left(\beta ,{X}_{j}\right)+{{a}_{i}+ e}_{ij}$$

where ${y}_{ij}$ is the weekly performance record (average daily growth rate) of individual chicken *i* in relation to the corresponding weekly mean or variance of weather variable* j*; *X* corresponds to a set of fixed effects including the sex of chicken and a combined batch number and local season effect (6 levels); $f\left(\beta ,{X}_{j}\right)$ is a covariate that corresponds to the population norm function describing the relationship between the average daily growth of all chickens and the corresponding mean or variance of weather variable *j* in the same week; ${a}_{i}$ corresponds to the deviation of individual chicken $i$ from the population average growth; and ${e}_{ij}$ corresponds to the random residual.

The function linking chicken growth to the weather variables in model (1) was a second-degree Legendre polynomial, implying a quadratic non-linear relationship between chicken growth profile and weather change. No genotypic information was included in this step and, therefore, population norms were expressed at the phenotypic level.

Model (1) equations were solved using the EM-REML algorithm implemented in the BLUPF90 suite of software [22] and produced estimates of the three coefficients (intercept, linear and quadratic) of the population norm.

### Individual deviation norms at the phenotypic level

A random regression model (2) was used to assess changes in individual chicken growth profiles in response to changing weather [21]:

2 $${y}_{ij}=X+f\left(\beta ,{X}_{j}\right)+{f}_{i}\left({a}_{i},{X}_{j}\right)+{e}_{ij}$$

where ${f}_{i}\left({a}_{i},{X}_{j}\right)$ corresponds to a second-degree Legendre polynomial function describing the relationship between the daily growth of chicken *i* and weather variable *j*, expressed as a deviation from the population norm $f\left(\beta ,{X}_{j}\right)$; all other effects are as in model (1). Again, as no genotypic information was included, individual deviation norms were expressed at the phenotypic level.

Solutions of the model (2) equations for the individual deviation coefficients (intercept, linear and quadratic) were derived using the EM-REML algorithm implemented in BLUPF90 [22]. Pearson correlations of the intercept with the linear and quadratic coefficients were estimated. Approximate standard errors of these correlations were also calculated as $(1-{r}^{2})\sqrt{(n-3)}$, where *r* is the correlation estimate and *n* is the sample size [23].

### Derivation of growth resilience phenotypes

Individual reaction norms were calculated by adding the individual deviations from model (2) to the corresponding population norm from model (1). One individual reaction norm was calculated for each chicken and the weekly mean of each weather variable, and another for each chicken and the weekly variance of the weather variable.

The points across a reaction norm curve correspond to chicken growth at the respective values of the weather variable. The slope of the tangent to the reaction norm at a given point of the curve represents the change in the chicken’s growth profile in response to changes in the specific weather value. This slope constitutes a potential growth resilience phenotype. Theoretically, there can be an infinite number of such phenotypes on each individual. For the purposes of the present study, we derived two resilience phenotypes for each chicken and individual reaction norm by calculating the derivatives at two points of the quadratic norm, each on either side of the absolute maximum value.

### Genomic analyses of resilience phenotypes

Variance component and heritability estimates of each growth resilience phenotype were derived with mixed model (3), using the GCTA software [24]:

3 $${\varvec{y}}={\varvec{W}}\boldsymbol{\alpha }+{\varvec{Z}}{\varvec{u}}+{\varvec{\varepsilon}}$$

where ***y*** represents the vector of growth resilience phenotypic values, ***W*** is the incidence matrix for vector ***α*** of fixed effects, ***Z*** is the incidence matrix for vector ***u*** of random polygenic effects (distributed as a multivariate normal distribution MVN(0,*Vg****G***) with ***G*** being the chicken genomic relatedness matrix and *Vg* the genomic variance of the trait), and ***ε*** represents the vector of residual errors (distributed as MVN(0,*Ve****I***) with ***I*** being the identity matrix and *Ve* the residual variance of the trait).

As the growth resilience phenotypes had already been corrected for the effects of sex of chicken, batch number and local season during the reaction norm calculation with models (1) and (2), there was no need to consider these effects again. However, we fitted the first three principal components (PC) derived from a PC analysis of the genotypes with software GCTA [24] as fixed effects in model (3) to account for any remaining population structure after including the genomic relatedness matrix ***G***.

The statistical significance of the variance component and heritability estimates derived with model (3) was assessed with the likelihood ratio test [25].

Subsequently, genome-wide association studies were performed on growth resilience phenotypes that had exhibited statistically significant (*P* < 0.05) genomic variance in the analysis with model (3). The following linear mixed model and GEMMA software [26] were used:

4 $${\varvec{y}}={\varvec{W}}\boldsymbol{\alpha }+{\varvec{x}}{\varvec{\beta}}+{\varvec{Z}}{\varvec{u}}+{\varvec{\varepsilon}}$$

where ***x*** represents the vector of genotypes at a SNP locus (coded as 0/1/2 according to the number of copies of the minor allele), ***β*** is the regression coefficient of the phenotype on the genotypes, and all other effects are as described in model (3).

A Bonferroni correction was applied for multiple testing to determine a genome-wide (*P* < 0.05) and suggestive (one false positive per genome scan) significance threshold, resulting in final threshold values of 1.7E-8 and 3.4E-7, respectively. The proportion of phenotypic variance explained by each of the identified significant SNPs was calculated as $[{2\beta }^{2}\times maf\times (1-maf)] / [{2\beta }^{2}\times maf\times (1-maf)+{\left(\text{se}\left(\beta \right)\right)}^{2}\times 2N\times maf\times (1-maf)]$, where *maf* was the SNP minor allele frequency, *β* and se(*β*) the SNP effect and standard error, and *N* the sample size [27]. Furthermore, annotated genes neighbouring the identified SNPs were investigated using the Ensembl database.

## Results

### Weather data exploration

Supplementary files 2 and 3 illustrate results from the exploratory analysis of seasonal variation of weather variables within and across years for the study period (2019–2021).

Air temperatures ranged between 4 and 33°C. Average daily temperatures were generally within the 15–20°C range but could dip lower during the cold local season (Bega) and rise higher during the hot local season (Belg). Minimum daily temperatures were relatively low, averaging below 10°C during the Bega local season. Maximum daily temperatures were usually mild, with an average below 25°C during the Bega and Kiremt local seasons but reaching higher values during the Belg season. Daily relative humidity was mostly in the range of 30%-60% during the Bega and Belg seasons but increased considerably during the wet local season (Kiremt), with values reaching over 75%. In terms of average daily precipitation, high values were relatively frequent during the Kiremt season, reflecting strong rainfalls (over 30 mm).

Table 2 summarises weekly means and variances of the studied weather variables corresponding to the weekly chicken growth measurements.

**Table 2:** Summary statistics for weekly means and variances of weather variables during the study period by local season

	**Maximum air temperature (Weekly mean of daily values)**				**Maximum air temperature (Weekly variance of daily values)**			
**Local Season**	**Minimum**	**Median**	**Mean**	**Maximum**	**Minimum**	**Median**	**Mean**	**Maximum**
**Bega**	22.20	23.57	23.49	25.02	0.18	0.98	1.06	3.02
**Belg**	23.37	25.47	25.54	27.25	0.69	1.20	1.94	5.60
**Kiremt**	19.77	20.98	21.10	22.47	0.30	1.55	1.62	3.19
	**Minimum air temperature (Weekly mean of daily values)**				**Minimum air temperature (Weekly variance of daily values)**			
**Local Season**	**Minimum**	**Median**	**Mean**	**Maximum**	**Minimum**	**Median**	**Mean**	**Maximum**
**Bega**	7.33	9.27	9.49	12.73	0.25	1.28	1.46	4.55
**Belg**	7.75	11.02	10.92	12.63	0.37	1.17	1.08	1.70
**Kiremt**	12.22	13.12	12.95	13.60	0.17	0.54	0.56	1.14
	**Average air temperature (Weekly mean of daily values)**				**Average air temperature (Weekly variance of daily values)**			
**Local Season**	**Minimum**	**Median**	**Mean**	**Maximum**	**Minimum**	**Median**	**Mean**	**Maximum**
**Bega**	14.70	16.00	16.11	17.28	0.05	0.38	0.43	1.41
**Belg**	15.87	17.72	17.32	18.27	0.25	0.29	0.66	2.47
**Kiremt**	15.45	16.25	16.20	17.22	0.12	0.37	0.42	0.75
	**Average relative humidity (Weekly mean of daily values)**				**Average relative humidity (Weekly variance of daily values)**			
**Local Season**	**Minimum**	**Median**	**Mean**	**Maximum**	**Minimum**	**Median**	**Mean**	**Maximum**
**Bega**	47.15	61.70	59.12	70.92	3.86	21.30	33.64	147.44
**Belg**	41.63	54.12	54.92	67.50	13.96	47.08	56.13	117.55
**Kiremt**	79.83	83.20	83.31	86.97	3.27	6.78	9.91	23.94
	**Average THI (Weekly mean of daily values)**				**Average THI (Weekly variance of daily values)**			
**Local Season**	**Minimum**	**Median**	**Mean**	**Maximum**	**Minimum**	**Median**	**Mean**	**Maximum**
**Bega**	14.63	15.69	15.73	16.66	0.03	0.22	0.26	0.88
**Belg**	15.39	17.01	16.57	17.25	0.07	0.11	0.33	1.41
**Kiremt**	15.37	16.04	16.02	16.92	0.09	0.28	0.37	0.60
	**Average precipitation (Weekly mean of daily values)**				**Average precipitation (Weekly variance of daily values)**			
**Local Season**	**Minimum**	**Median**	**Mean**	**Maximum**	**Minimum**	**Median**	**Mean**	**Maximum**
**Bega**	0.00	0.05	0.37	3.45	0.00	0.01	3.53	58.15
**Belg**	0.00	0.00	0.24	1.38	0.00	0.00	0.45	2.59
**Kiremt**	3.00	8.97	9.63	16.97	4.91	114.63	133.47	353.67

Temperatures are in degrees centigrade (^o^C), relative humidity in percentage and amount of precipitation in millimetres

*THI* Temperature-humidity index

### Population reaction norms

**Fig. 1:**
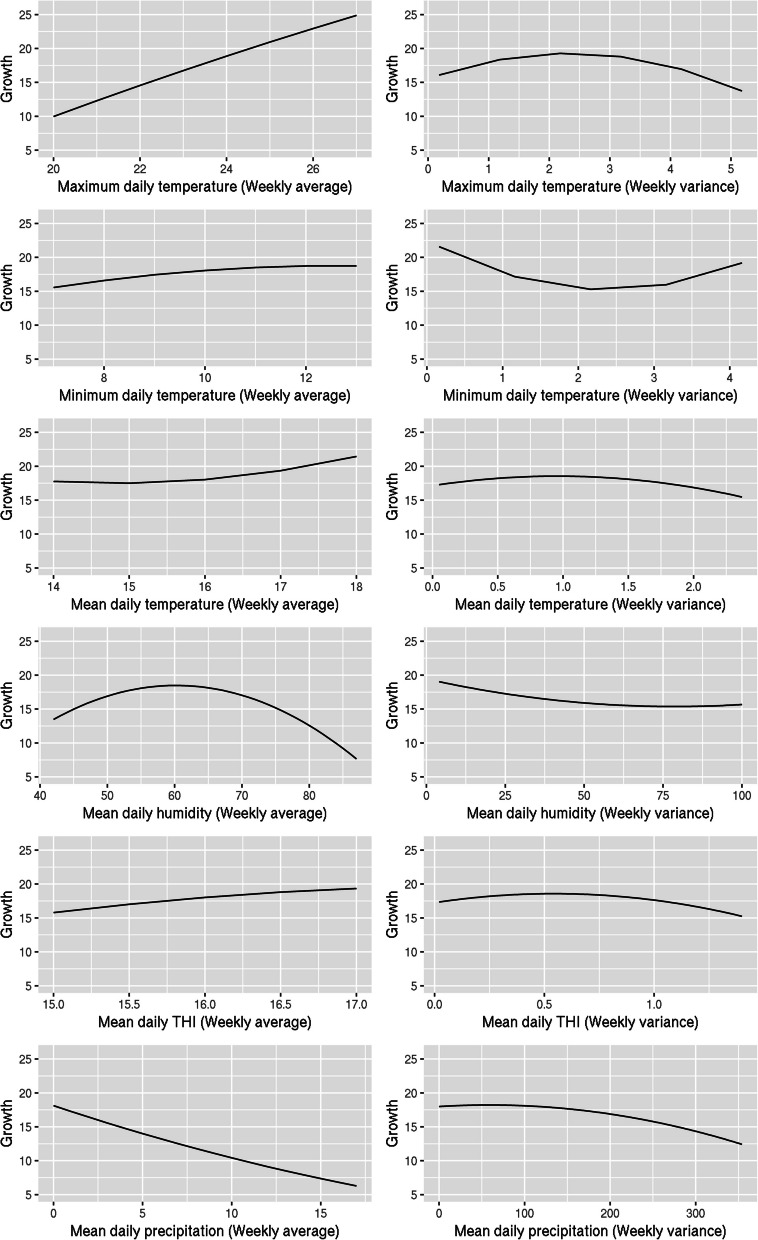
Population reaction norms at the phenotypic level for each weather variable. Air temperatures are in degrees centigrade (^o^C), relative humidity in percentage, and amount of precipitation in millimetres (mm); growth is in grams per day

Estimates of the intercept, linear and quadratic coefficients of the population norm are presented in Table 3. Population norms for each weather variable are illustrated in Fig. 1.

**Table 3:** Estimates and standard errors of the population norm coefficients by weather variable

		a_0_ (SE)	a_1_ (SE)	a_2_ (SE)
Weekly mean	Maximum daily temperature	10.25 (0.44)	7.46 (0.41)	-0.27 (0.28)
	Minimum daily temperature	13.75 (0.41)	1.60 (0.28)	-0.61 (0.21)
	Average daily temperature	12.51 (0.45)	1.84 (0.25)	1.05 (0.30)
	Average daily humidity	12.10 (0.43)	-2.92 (0.40)	-5.10 (0.35)
	Average daily THI	13.27 (0.46)	1.78 (0.24)	-0.31 (0.34)
	Average daily precipitation	8.24 (1.04)	-5.92 (0.61)	0.51 (0.43)
Weekly variance	Maximum daily temperature	14.01 (0.41)	-1.17 (0.28)	-2.87 (0.22)
	Minimum daily temperature	14.45 (0.40)	-1.19 (0.25)	3.39 (0.21)
	Average daily temperature	14.58 (0.42)	-0.91 (0.29)	-1.38 (0.25)
	Average daily humidity	14.17 (0.38)	-1.69 (0.25)	1.01 (0.21)
	Average daily THI	14.74 (0.43)	-1.05 (0.29)	-1.44 (0.26)
	Average daily precipitation	13.26 (0.59)	-2.78 (0.29)	-1.39 (0.29)

a_o_, a_1_, a_2_ refer to the intercept, linear, and quadratic terms, respectively

All linear coefficients and most quadratic coefficients were significantly different from zero, thereby reflecting a significant effect of the corresponding weather variable on chicken growth. Maximum air temperature, average precipitation and average relative humidity had the strongest effect on the overall chicken growth profile change.

### Individual deviation reaction norms

Table 4 presents summary statistics of the intercept, linear, and quadratic coefficients of the individual deviation norms. The arithmetic means of these coefficients are not included as they were practically zero, since they represented the individual chicken deviation from the population norm.

**Table 4:** Estimates and summary statistics of the coefficients for the individual deviation norms by weather variable

		**a**_**0**_			**a**_**1**_			**a**_**2**_		
		**M**	**SEM**	**σ**	**M**	**SEM**	**σ**	**M**	**SEM**	**σ**
**Weekly mean**	**Maximum daily temperature**	0.058	0.039	1.564	-0.001	0.006	0.230	-0.024	0.011	0.423
	**Minimum daily temperature**	0.061	0.039	1.541	-0.015	0.008	0.322	0.004	0.004	0.159
	**Average daily temperature**	0.050	0.038	1.526	-0.002	0.001	0.042	-0.060	0.016	0.648
	**Average daily humidity**	0.045	0.034	1.366	-0.064	0.021	0.847	-0.003	0.007	0.273
	**Average daily THI**	0.043	0.040	1.582	0.004	0.001	0.055	-0.038	0.009	0.377
	**Average daily precipitation**	0.054	0.038	1.511	-0.013	0.011	0.456	-0.013	0.008	0.338
**Weekly variance**	**Maximum daily temperature**	0.061	0.043	1.700	0.005	0.003	0.114	-0.002	0.002	0.079
	**Minimum daily temperature**	0.057	0.041	1.637	-0.002	0.002	0.075	0.017	0.009	0.348
	**Average daily temperature**	0.062	0.043	1.705	0.004	0.003	0.100	-0.007	0.005	0.205
	**Average daily humidity**	0.048	0.040	1.594	-0.001	< 0.001	0.017	< 0.001	< 0.001	< 0.001
	**Average daily THI**	0.062	0.041	1.652	0.002	0.001	0.044	-0.006	0.004	0.170
	**Average daily precipitation**	0.040	0.036	1.443	-0.007	0.005	0.214	< 0.001	0.001	0.021

a_o_, a_1_, a_2_ are intercept, linear, and quadratic terms, respectively; summary statistics are median (M), standard error of the mean (SEM) and standard deviation (σ) calculated across all individuals

Correlations of the intercept with the linear and quadratic coefficients are shown in Table 5. These estimates were significantly different from zero (*P* < 0.05).

**Table 5:** Product-moment correlations and their standard errors between the coefficients of the individual deviation norms by weather variable

		**r**_**a0_a1**_** (SE)**	**r**_**a0_a2**_** (SE)**
**Weekly mean**	**Maximum daily temperature**	0.79 (0.01)	-0.96 (< 0.02)
	**Minimum daily temperature**	-0.98 (< 0.01)	0.98 (< 0.01)
	**Average daily temperature**	-0.25 (0.02)	0.06 (0.03)
	**Average daily humidity**	-0.51 (0.02)	-0.87 (0.01)
	**Average daily THI**	-0.72 (0.01)	0.62 (0.02)
	**Average daily precipitation**	-1.00 (< 0.01)	-1.00 (< 0.01)
**Weekly variance**	**Maximum daily temperature**	1.00 (< 0.01)	-1.00 (< 0.01)
	**Minimum daily temperature**	0.52 (0.02)	0.97 (0.01)
	**Average daily temperature**	1.00 (< 0.01)	-1.00 (< 0.01)
	**Average daily humidity**	-0.16 (0.03)	-0.84 (0.01)
	**Average daily THI**	0.98 (< 0.01)	-1.00 (< 0.01)
	**Average daily precipitation**	-1.00 (< 0.01)	-0.86 (0.01)

r_a0_a1_ and r_a0_a2_ correspond to the correlations between the intercept and the linear term and between the intercept and the quadratic term, respectively

### Resilience phenotypes

Table 6 presents summary statistics for the chicken growth resilience phenotypes to each weather variable developed in the present study.

**Table 6:** Summary statistics for the growth resilience phenotypes by weather variable

		**P**	**˄**	**˅**	**M**	**µ** (**SE)**	**σ**	**CV%**
**Weekly mean**	**Maximum daily temperature**	22 (^o^C)	1.52	2.82	2.21	2.20 (0.01)	0.20	9.2
		26 (^o^C)	1.23	2.79	1.99	1.99 (0.01)	0.23	11.4
	**Minimum daily temperature**	8 (^o^C)	0.33	1.67	0.95	0.95 (0.01)	0.21	21.9
		11 (^o^C)	0.12	0.59	0.34	0.34 (< 0.01)	0.06	18.3
	**Average daily temperature**	15 (^o^C)	-1.97	3.55	0.30	0.26 (0.01)	0.47	182.7
		17 (^o^C)	-2.10	3.83	1.40	1.45 (0.01)	0.50	34.8
	**Average daily humidity**	50 (%)	0.16	0.48	0.31	0.31 (0.001)	0.04	14.2
		70 (%)	-0.43	-0.14	-0.29	-0.29 (0.001)	0.04	13.5
	**Average daily THI**	15.5	-0.54	6.22	2.50	2.44 (0.02)	0.62	25.4
		16.5	-2.07	3.50	1.05	1.10 (0.01)	0.51	46.6
	**Average daily precipitation**	3 (mm)	-0.89	-0.67	-0.79	-0.79 (0.01)	0.03	3.3
		13 (mm)	-0.96	-0.23	-0.62	-0.61 (0.01)	0.12	19.0
**Weekly variance**	**Maximum daily temperature**	1.18 (^o^C)	1.26	1.93	1.63	1.62 (< 0.01)	0.10	6.3
		4.18 (^o^C)	-2.59	-2.47	-2.53	-2.53 (< 0.01)	0.01	0.5
	**Minimum daily temperature**	1.16 (^o^C)	-3.87	-2.40	-3.19	-3.18 (< 0.01)	0.24	7.4
		3.16 (^o^C)	0.84	2.96	1.97	1.96 (< 0.01)	0.29	14.8
	**Average daily temperature**	0.53 (^o^C)	-0.06	2.43	1.27	1.26 (0.01)	0.40	31.6
		1.53 (^o^C)	-1.89	-1.49	-1.71	-1.70 (< 0.01)	0.06	3.6
	**Average daily humidity**	35 (%)	-0.06	-0.06	-0.06	-0.06 (< 0.01)	< 0.01	0.6
		95 (%)	0.02	0.03	0.02	0.02 (< 0.01)	< 0.01	3.1
	**Average daily THI**	0.4	-0.06	2.40	1.24	1.23 (0.01)	0.40	32.2
		1.2	-7.13	-4.19	-5.75	-5.76 (0.01)	0.46	8.1
	**Average daily precipitation**	100 (mm)	-0.01	-0.00	-0.01	-0.01 (< 0.01)	< 0.01	20.3
		250 (mm)	-0.03	-0.02	-0.03	-0.03 (< 0.01)	< 0.01	5.3

Resilience phenotypes pertain to specific points (P) on the population norm curve of the corresponding weather variable; summary statistics include minimum (˄), maximum (˅), Median (M), mean (μ) and standard error (SE), standard deviation (σ), and coefficient of variation (CV, expressed in percentage)

The resilience phenotypes associated with the weekly means of average daily temperature and THI change exhibited the highest variability, manifested in the corresponding coefficient of variation estimates. The lowest variation was mainly observed for resilience phenotypes associated with weekly variances of the weather variables.

### Genomic analyses of resilience phenotypes

The variance component and heritability estimates of the growth resilience phenotypes are shown in Table 7.

**Table 7:** Variance component and heritability estimates and standard errors (SE) for the growth resilience phenotypes by weather variable

		**P**	**V**_**G**_	**SE(V**_**G**_**)**	**V**_**E**_	**SE(V**_**E**_**)**	**h**^**2**^	**SE(h**^**2**^**)**	***P*-value**
**Weekly mean**	**Maximum daily temperature**	22 (^o^C)	0.003	0.003	0.038	0.003	0.07	0.07	0.16
		26 (^o^C)	< 0.001	0.003	0.051	0.004	0.01	0.07	0.45
	**Minimum daily temperature**	8 (^o^C)	0.004	0.003	0.040	0.003	0.09	0.07	0.11
		11 (^o^C)	< 0.001	< 0.001	0.004	< 0.001	0.11	0.07	0.05*
	**Average daily temperature**	15 (^o^C)	0.032	0.015	0.181	0.014	0.15	0.07	0.01*
		17 (^o^C)	0.037	0.017	0.210	0.017	0.15	0.07	0.01*
	**Average daily humidity**	50 (%)	< 0.001	< 0.001	0.002	< 0.001	< 0.01	0.06	0.50
		70 (%)	< 0.001	< 0.001	0.002	< 0.001	< 0.01	0.07	0.50
	**Average daily THI**	15.5	0.077	0.028	0.300	0.026	0.21	0.07	< 0.01*
		16.5	0.055	0.019	0.203	0.018	0.21	0.07	< 0.01*
	**Average daily precipitation**	3 (mm)	< 0.001	< 0.001	0.001	< 0.001	0.05	0.07	0.21
		13 (mm)	0.001	< 0.001	0.013	0.001	0.08	0.07	0.11
**Weekly variance**	**Maximum daily temperature**	1.18 (^o^C)	0.001	< 0.001	0.010	0.001	0.10	0.07	0.09
		4.18 (^o^C)	< 0.001	< 0.001	< 0.001	< 0.001	< 0.01	0.07	0.50
	**Minimum daily temperature**	1.16 (^o^C)	0.004	0.004	0.052	0.004	0.07	0.07	0.16
		3.16 (^o^C)	0.006	0.006	0.078	0.003	0.07	0.07	0.16
	**Average daily Temperature**	0.53 (^o^C)	0.015	0.011	0.144	0.011	0.097	0.07	0.09
		1.53 (^o^C)	< 0.001	< 0.001	0.004	< 0.001	0.067	0.07	0.17
	**Average daily humidity**	35 (%)	< 0.001	< 0.001	< 0.001	< 0.001	0.057	0.07	0.22
		95 (%)	< 0.001	< 0.001	< 0.001	< 0.001	0.007	0.07	0.50
	**Average daily THI**	0.4	0.014	0.011	0.143	0.011	0.097	0.07	0.09
		1.2	0.017	0.015	0.199	0.015	0.08	0.07	0.12
	**Average daily precipitation**	100 (mm)	< 0.001	< 0.001	< 0.001	< 0.001	0.097	0.07	0.09
		250 (mm)	< 0.001	< 0.001	< 0.001	< 0.001	0.087	0.07	0.12

Resilience phenotypes pertain to specific points (P) on the population norm curve of the corresponding weather variable; parameters include the genomic variance (V_G_), residual variance (V_E_), heritability (h^2^) and corresponding standard errors (SE); P-values refer to heritability estimates. Significant *P*-values (*P* < 0.05) from the Likelihood Ratio Test are indicated with *

Statistically significant genomic variance estimates (*P* < 0.05) were derived for five growth resilience phenotypes pertaining to the weekly means of average daily air temperature, minimum daily temperature, and THI. Corresponding heritability estimates for these traits were moderately low, ranging from 0.11 to 0.21. The other resilience phenotypes did not exhibit significant genomic variation (*P* > 0.05).

No inflation was detected in the ensuing genome-wide association study, with the lambda factor values ranging from 0.998 to 1.003. No genome-wide significant SNPs were detected for any of the studied phenotypes. However, three genome-wide suggestive SNPs were found for the growth resilience phenotypes related to weekly mean THI and another two suggestive SNPs were associated with response to the weekly mean of minimum daily temperature (Table 8). In all cases, multiple trailing SNPs supported the corresponding hits relatively well (Supplementary Files 4, 5 and 6). Two of the former SNPs were common to both THI resilience phenotypes at the two points of the reaction norm. The proportion of variance explained by each SNP is included in Table 8.

**Table 8:** Suggestive single nucleotide polymorphisms detected for growth resilience phenotypes

		**P**	**Chr**	**Position (in bp)**	**Beta effect and standard error**	**MAF**	**P-value**	**PVE (%)**
**Weekly mean**	**Minimum temperature**	11 (^o^C)	4	23,738,216	0.013 ± 0.002	0.468	1.2E-7	1.75
		11 (^o^C)	3	107,013,067	0.039 ± 0.007	0.025	1.5E-7	1.72
	**Average THI**	15.5	3	43,321,960	-0.132 ± 0.025	0.384	1.5E-7	1.73
		15.5	1	4,752,206	0.393 ± 0.075	0.022	1.9E-7	1.70
		16.5	3	43,321,960	0.109 ± 0.021	0.384	1.6E-7	1.67
		16.5	1	4,752,206	-0.320 ± 0.062	0.022	2.7E-7	1.65
		16.5	2	39,779,728	0.290 ± 0.056	0.028	2.9E-7	1.66

Resilience phenotypes pertain to specific points (P) on the population norm curve of the corresponding weather variable. Chr, MAF and PVE correspond to the chromosome number, minor allele frequency and the percentage of phenotypic variance explained by the marker, respectively

## Discussion

The present study aimed to derive and examine individual growth resilience phenotypes to weather changes of commercial chickens reared in smallholder farm conditions in Ethiopia and to investigate the genomic architecture of these new phenotypic traits.

### Weather variable profiles

The venue of the study was the ILRI poultry research facility in Addis Ababa, Ethiopia, which is located in an elevated region at 2,382 m above sea level with a weather profile reflective of the Ethiopian highlands. The study took place across three distinct local seasons: Belg, Bega and Kiremt. The Belg local season (February to May) was characterised by the highest maximum daily temperatures, often exceeding 25°C but rarely reaching 30°C. This season also presented the lowest average daily humidity, with values commonly in the range of 40% to 60%. The Bega local season (October to January) featured the lowest minimum daily temperatures, with values usually under 10°C, and a relative humidity only slightly higher than the one observed in Belg. Both Belg and Bega were generally dry seasons with very little precipitation in the form of rain. On the contrary, the Kiremt local season (June to September) was characterised by high daily relative humidity and considerable precipitation levels. However, air temperatures were milder than in the other two seasons.

While the prevailing air temperatures in the region can be considered moderate, the relatively short range of the chicken thermo-neutrality zone (18–24°C) [28, 29] implies the possibility of observing effects of both heat stress and, perhaps more likely, cold stress in the chickens of study. The impact of relative humidity is not well known, with most studies indicating that values ranging from 25 to 75% play a minor role, if any, unless accompanied by high temperatures [30, 31]. In the present study, the latter was captured in the calculated THI. Similarly, strong precipitation can be related to an increase in the transmission of parasitic diseases such as coccidiosis, thereby affecting chicken growth [32].

### Reaction norms and growth resilience phenotypes to weather change

Intercepts and linear coefficients of the population reaction norms were always significantly different from zero (*P* < 0.05). The signs of the linear coefficients meant that chicken growth generally benefitted from increasing air temperature and THI and decreasing humidity and precipitation. The negative signs of the linear coefficients associated with the weekly variance of the weather variables suggested that growth was challenged by increased weather instability. The quadratic coefficients were also statistically significant (*P* < 0.05) except for the weekly mean of maximum air temperature, THI, and amount of precipitation. When significant, this coefficient revealed a non-linear reaction of average chicken growth to changing weather events. In case of the latter, the direct numerical interpretation of some of these results can be challenging. A linear coefficient represents the expected change in growth per unit change in a weather variable, and in the absence of a quadratic term, this rate of change remains constant across the entire range of weather values. However, a significant quadratic coefficient indicates the convex or concave nature of the relationship, suggesting that the rate of change is not uniform across the range of values. For example, a 1°C increase in average daily temperature was associated with an increase in average daily growth of 1.84 g within the studied range of 14–18°C. The significant quadratic coefficient of 1.05, however, indicated a convex relationship, where growth only increased when temperatures exceeded 15.5°C. In contrast, the interpretation of the effects of THI and precipitation were more straightforward, as the quadratic terms were not significant. For these variables, a unit increase within their mean value ranges (15–17 and 0–17 mm, respectively) resulted in a daily growth increase of 1.78 g for THI and a decrease of 5.92 g for precipitation.

For the individual deviation norms, the linear coefficient on average relative humidity and the quadratic coefficient on average air temperature exhibited the highest variance, suggesting that individual chickens may respond differently to these weather effects. The variation of the linear and quadratic coefficients on the weekly variances of weather events was low, implying that most chickens may respond similarly to this challenge.

Exploring the correlations of the intercept with the linear and quadratic coefficients of the individual deviation norms provided an insight into the possible phenotypic relationship between the actual level of chicken growth (intercept) and changes in growth profile in response to weather fluctuations (linear and quadratic coefficients). In the present study, all these phenotypic correlations were significantly different from zero (P < 0.05). For the weekly means of weather variables, negative linear and positive quadratic correlations were found for minimum and average air temperature and THI, suggesting that fast growing chickens may be relatively resilient at low values of these variables but quickly become adversely affected as values increase. This result complements the positive linear and negative quadratic correlation observed for maximum air temperature, implying that fast growing chickens may benefit from low values but the benefit diminishes quickly as air temperature increases. Conversely, slow growing chickens seem to be more resilient to high temperatures, but also more vulnerable to the cold. These results are in line with the expectation that small chickens are more susceptible to stress due to low air temperature, as their high surface area compared to volume may lead to faster rates of heat loss [33]. Similarly, fast growing chickens are expected to be more susceptible to heat stress [34], but the range of maximum air temperatures observed in the present study was moderate and only slightly over the thermo-neutral zone of chickens. Thus, although we observe a diminishing benefit of high temperatures for fast growing chickens, we still do not see the full adverse effect of heat stress on growth.

Negative linear and negative quadratic correlations for relative humidity and amount of precipitation indicate that fast growing chickens are likely to be more affected by increasing values of these weather variables, implying higher humidity and heavy rain, than slowly growing chickens.

The correlation estimates pertaining to weekly variances of weather variables collectively suggested that fast growing chickens might generally be less susceptible to variation in daily air temperature, but more susceptible to variation in humidity and precipitation. We speculate that, while larger chickens may tolerate weekly weather variation, particularly during short-term cold stress events, they face potential difficulties when looking for shelter under sporadic heavy precipitation. Also, reduced feed intake while sheltering and not scavenging may have a greater impact on larger birds with higher metabolic demand.

Notably, the associations between chicken growth and resilience manifested in all these correlations are at the phenotypic level, and future research should investigate their genetic component.

Novel phenotypes of chicken growth resilience were derived here based on the reaction norm coefficients, reflecting changes in individual chicken growth profiles in response to changing weather conditions. Large variations in resilience would suggest that individual chickens react differently to this environmental challenge. We observed the largest phenotypic variation in growth resilience in response to average air temperature and THI changes. Interestingly, these weather variables exhibited relatively small fluctuations across the timeline of the study, manifested in the low coefficient values in the respective population norm. As such, it is important to clarify that a significant population norm coefficient may indicate an overall strong effect of the weather variable on growth, but it does not necessarily imply variation in the individual resilience phenotypes. Similarly, there is a distinction between variation in the coefficients of the individual deviation norms and variation in the respective resilience phenotypes, as the former pertains to the entire curve of the norm while the latter is estimated at specific points of the curve.

### Genomic analyses of growth resilience phenotypes

Significant genomic variance and heritability estimates were derived for growth resilience to changes in average air temperature, minimum air temperature and THI. These estimates ranged from 0.11 to 0.21 and are considered moderately low, in line with previous studies on fitness-related traits in chickens [35] and other species [36, 37]. Despite the low heritability, the presence of genomic variation among individual chickens supports the possibility of selective breeding to enhance growth resilience within future breeding programmes [38].

Genome-wide association analyses revealed five SNP markers that were suggestively associated with the growth resilience phenotypes that exhibited significant genomic variance.

The SNP identified on chromosome 4 in association with growth resilience to changes in minimum air temperature was located within the region of gene *ENSGALG00000047398*, a long non-coding RNA (lncRNA). In recent years, several studies have identified lncRNAs as major players in the regulation of gene expression in biological networks associated with diverse processes [39], including metabolic response to heat and cold stress in both plants [40, 41] and animals [42, 43]. These processes also include the regulation of adipose tissue development and activity [44].

The SNP on chromosome 3, also associated with growth resilience to changes in minimum air temperature, was not found within any coding region but was in linkage disequilibrium (within 200 Kb) with the *PINX1* gene, which codifies for a telomerase inhibitor. Telomerase inhibitors prevent telomere elongation and help maintain telomere homeostasis [45]. Air temperature stress is known to affect telomeric integrity [46] and previous studies have associated leucocyte telomere attrition rates with weather conditions and productivity in other farm animal species [47]. It is plausible that telomerase inhibitors play a significant role in the resilience of animal production to weather change through an effect on telomere integrity and cell senescence.

The SNP on chromosome 3 associated with THI change is located within the coding region of the *phosphodiesterase 10A* gene (*PDE10A*). Phosphodiesterases are involved in the regulation of cyclic AMP and cyclic GMP in multiple processes [48]. Previous studies linked phosphodiesterase 10A with osteogenic differentiation [49], lung inflammation [50] and vascular remodelling [51]. Furthermore, it has been proposed that the inhibition of the enzyme may protect the individual from diet-induced obesity and insulin resistance [52]. Interestingly, studies in mice [53, 54] implicated *PDE10A* in thermoregulation and obesity: inhibition of phosphodiesterase 10A appeared to stimulate thermogenic gene expression and reduce hedonic feeding. Thus, *PDE10A* could possibly be related to energy homeostasis in response to weather change.

The SNP on chromosome 1 associated with THI change was found within the coding region for the *inter-alpha-trypsin inhibitor heavy chain* (*ITIH5*). This protein belongs to a family of plasma inhibitors that have an important role in inflammation and carcinogenesis [55]. *ITIH5* reportedly inhibits the growth and metastasis of melanoma in humans [56]. A recent study [57] linked *ITIH5* with the regulation of adipose tissue homeostasis, which may suggest a role in the coping mechanism to environmental stress, particularly considering that the non-shivering thermogenesis of the brown adipose tissue may be activated by cold stimulation [58]. Furthermore, genes involved in lipid metabolism are reportedly highly enriched under heat stress in the avian liver [59], where most of the lipogenesis occur [60]. These genes may also contribute to chicken response to varying weather conditions. Finally, *ITIH5* is a p-53 responsive gene [56], and heat stress can induce apoptosis by activating p-53 mediated mitochondrial pathways [61]. This indicates that *ITIH5* may have a role in cell response to weather stressors.

The SNP on chromosome 2, associated with growth resilience to changes in THI, is located within the coding region for *TGFβR2* (*transforming growth factor beta receptor 2*). A differential expression of this growth factor was previously identified in a study related to heat stress of Holstein cows and apoptosis processes in fibroblasts [62]. Furthermore, as previously mentioned, heat stress can induce apoptosis by activating p-53 mediated pathways, and the inactivation of p-53 is also known to distort TGFβ signalling and promote tumour growth and malignancy [63].

Noticeably, the SNPs on chromosomes 1 and 3 affected resilience to THI change at both sides of the reaction norm and the effect had the opposite sign in the two cases. This is not necessarily surprising, as response to stress is highly dependent on gene expression, with different patterns activating and deactivating metabolic networks [64].

The proportion of variance explained by each SNP was between 1.65% and 1.73%, within the range of previous reports on other chicken traits [65, 66], although our estimates might be slightly inflated due to the Beavis effect [67]. This result, together with the overall illustration of all individual SNP effects (Supplementary Files 4, 5 and 6) suggest a largely polygenic architecture for the studied resilience phenotypes. This is typical for fitness-related traits [68], where multiple genes, each contributing a relatively modest effect, collectively control the genetic background and influence the resilience phenotypes under investigation. Selective breeding based on estimated genomic breeding values is the best approach to genetic enhancement of chicken performance for such traits [69]. Nevertheless, results on significant genetic variants may be incorporated into the genomic evaluation process to increase the accuracy of the ensuing selection [70].

Finally, research is warranted on the biological mechanisms through which weather fluctuations influence growth, including heat stress, immunosuppression, and reduced feed intake, as well as the potential interactions among them. Given their complexity, chicken resilience to weather change becomes a truly multifactorial trait, beyond the polygenic architecture reported in the present study. Thus, a deeper understanding of the underlying biological mechanisms is crucial for improving the accuracy of predictive models and further refining the discovery of relevant genomic variants. Other environmental factors, particularly those experienced during incubation and early chick development, may also play a role in shaping future thermal resilience. While batch effects in the present study accounted for these conditions to some extent, there may still be unexplained variation affecting the physiological response to environmental stress. Furthermore, seasonal variations in disease prevalence add yet another layer of complexity, affecting chicken performance independently of weather fluctuations. Recognising these multifaceted influences is essential for a holistic analytical approach towards understanding the specific mechanisms of environmental resilience.

## Conclusions

To our knowledge, this is the first phenotypic and genetic study of growth resilience in commercial chickens in Ethiopia. We have shown that random regression models can be used to derive resilience phenotypes describing changes in the chicken growth profiles in response to varying weather conditions. The significant genomic variance and candidate genes reported here may be used to inform breeding programmes aimed at addressing climate change and sustainably improving chicken production under ever-evolving environmental challenges. Future studies should investigate the genetic correlation of the new growth resilience phenotypes with other chicken traits in the breeding goals. Furthermore, additional studies should focus on other chicken ecotypes in sub-Saharan Africa, including local breeds, to reveal possible distinctive adaptation patterns.

## Supplementary Information

Supplementary Material 1.

Supplementary Material 2.

Supplementary Material 3.

Supplementary Material 4.

Supplementary Material 5.

Supplementary Material 6.

## Data Availability

The datasets generated and/or analysed during the current study are available in the University of Edinburgh repository (Edinburgh DataShare), https://datashare.ed.ac.uk/handle/10283/8761.
