# Supplementary material for: Growth resilience to weather variation in commercial free-ranging chickens in Ethiopia
Source: BMC Genomics. 2025 Apr 14;26:371. doi: 10.1186/s12864-025-11561-6 (PMC11998408; doi:10.1186/s12864-025-11561-6)
Supplement: Supplementary file 1 — Supplementary Material 1. [file 12864_2025_11561_MOESM1_ESM.docx]

**Supplementary File 1.docx:** Meteorological stations providing weather data in Addis Ababa.

| **Station name** | **Station Identifier** | **Distance (km)** | **Latitude (DD)** | **Longitude (DD)** |
| --- | --- | --- | --- | --- |
| HAAB | HAAB | 7.3 | 8.99 | 38.79 |
| BOLE INTERNATIONAL, ET | 63450099999 | 8.9 | 8.978 | 38.799 |
| HARAR MEDA MIL, ET | 63451099999 | 43.7 | 8.733 | 39 |

Distance to the study site and geographic coordinates of the stations in decimal degrees (DD).
