# Supplementary material for: Growth resilience to weather variation in commercial free-ranging chickens in Ethiopia
Source: BMC Genomics. 2025 Apr 14;26:371. doi: 10.1186/s12864-025-11561-6 (PMC11998408; doi:10.1186/s12864-025-11561-6)

**Supplementary File 2.docx: Distributions of daily weather variables by local season across the calendar years 2019-2021**. Air temperatures are in degrees centigrade (^o^C), relative humidity in percentage, and amount of precipitation in millimetres (mm).


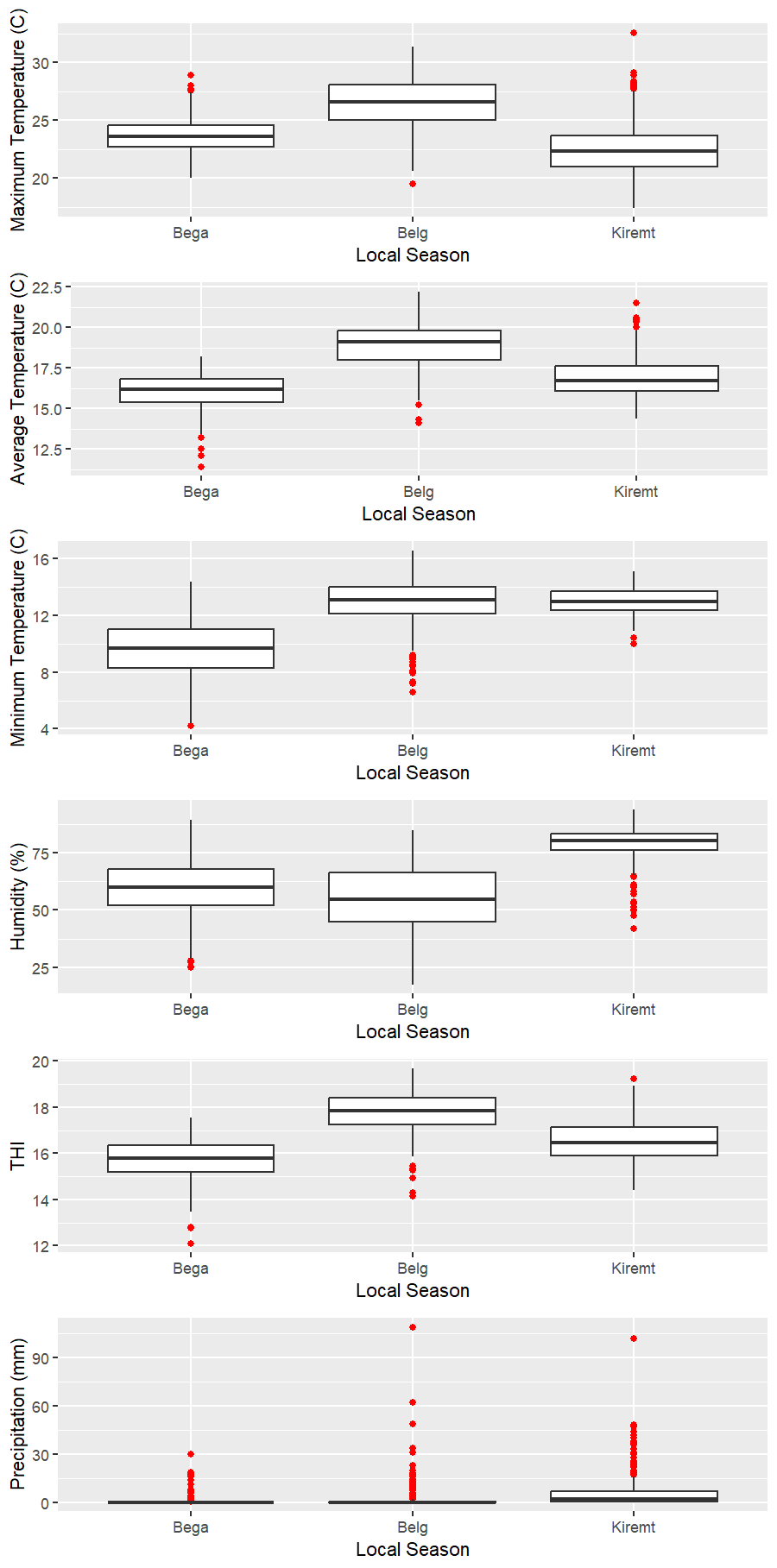

Supplement: Supplementary file 2 — Supplementary Material 2. [file 12864_2025_11561_MOESM2_ESM.docx]
