# Supplementary material for: Growth resilience to weather variation in commercial free-ranging chickens in Ethiopia
Source: BMC Genomics. 2025 Apr 14;26:371. doi: 10.1186/s12864-025-11561-6 (PMC11998408; doi:10.1186/s12864-025-11561-6)

**Supplementary File 4.docx:** Manhattan plots related to growth resilience phenotypes to minimum temperature at 11^o^C for chromosomes 4 and 3. Genome-wide significance threshold is in red and genome-wide suggested threshold in blue. Potential genes of interest associated with the identified SNPs were *ENSGALG00000047398* (chromosome 4) and *PINX1* (chromosome 3).


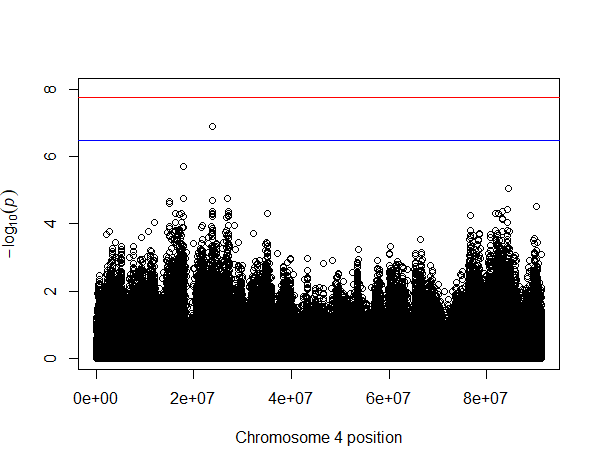


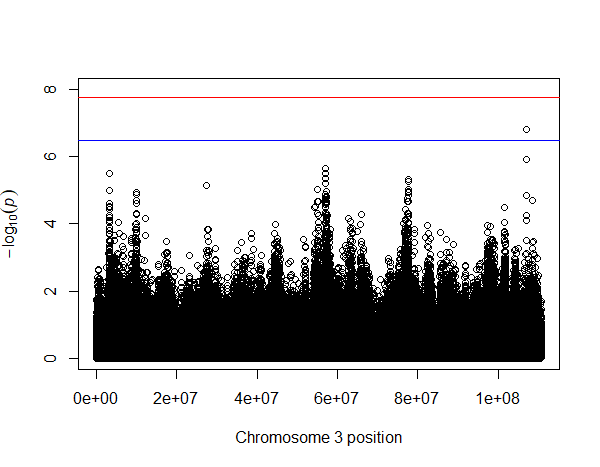

Supplement: Supplementary file 4 — Supplementary Material 4. [file 12864_2025_11561_MOESM4_ESM.docx]
