# Supplementary material for: Growth resilience to weather variation in commercial free-ranging chickens in Ethiopia
Source: BMC Genomics. 2025 Apr 14;26:371. doi: 10.1186/s12864-025-11561-6 (PMC11998408; doi:10.1186/s12864-025-11561-6)

**Supplementary File 5.docx:** Manhattan plots related to growth resilience phenotypes to THI at 15.5 for chromosomes 3 and 1. Genome-wide significance threshold is in red and genome-wide suggested threshold in blue. Potential genes of interest associated with the identified SNPs were *PDE10A* (chromosome 3) and *ITIH5* (chromosome 1).


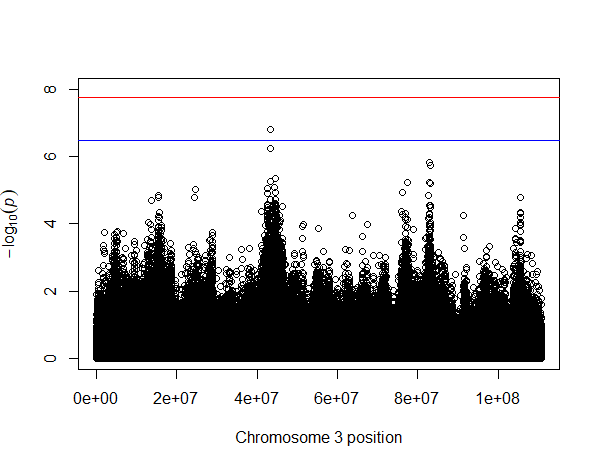


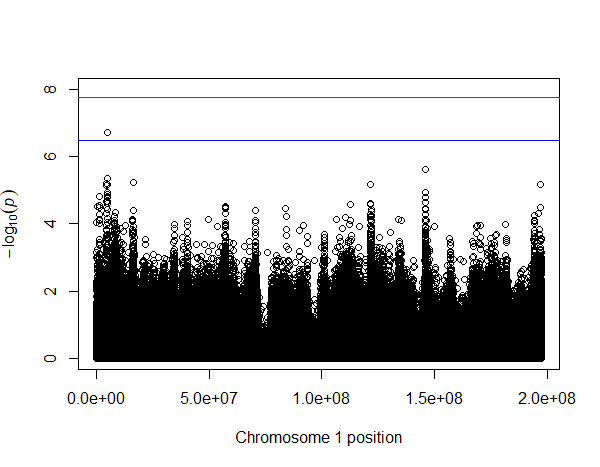

Supplement: Supplementary file 5 — Supplementary Material 5. [file 12864_2025_11561_MOESM5_ESM.docx]
