# Supplementary material for: Growth resilience to weather variation in commercial free-ranging chickens in Ethiopia
Source: BMC Genomics. 2025 Apr 14;26:371. doi: 10.1186/s12864-025-11561-6 (PMC11998408; doi:10.1186/s12864-025-11561-6)

**Supplementary File 6.docx:** Manhattan plots related to growth resilience phenotypes to THI at 16.5 for chromosomes 3, 1 and 2. Genome-wide significance threshold is in red and genome-wide suggested threshold in blue. Potential genes of interest associated with the identified SNPs were *PDE10A* (chromosome 3), *ITIH5* (chromosome 1) and *TGFβR2* (chromosome 2).


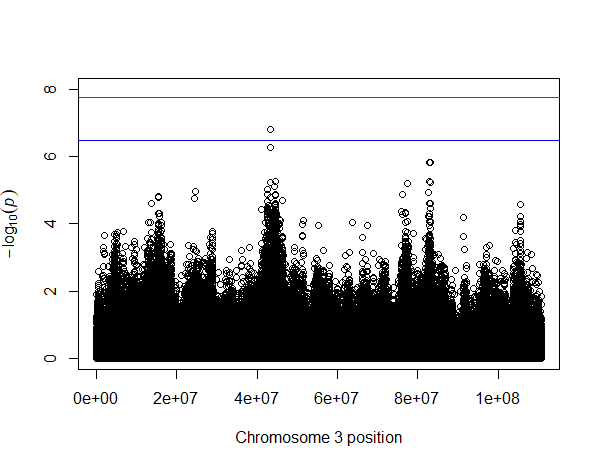

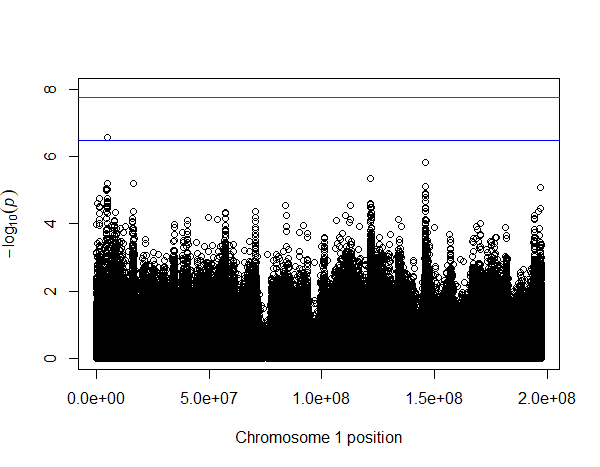

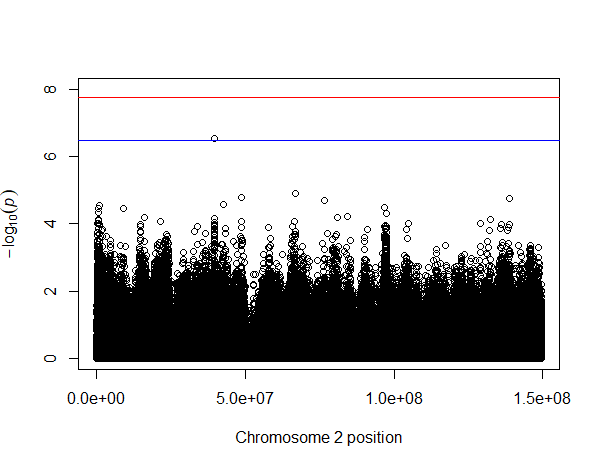

Supplement: Supplementary file 6 — Supplementary Material 6. [file 12864_2025_11561_MOESM6_ESM.docx]
